# A multi-center, randomized, double-blind, parallel, placebo-controlled trial to evaluate the efficacy, safety, and pharmacokinetics of intravenous ibuprofen for the treatment of fever in critically ill and non-critically ill adults

**DOI:** 10.1186/cc9089

**Published:** 2010-06-30

**Authors:** Peter E Morris, John T Promes, Kalpalatha K Guntupalli, Patrick E Wright, Murray M Arons

**Affiliations:** 1 Pulmonary-Critical Care, Wake Forest University School of Medicine, Winston Salem, NC 27157, USA; 2 Department of Surgery, Orlando Regional Medical Center, 77 West Underwood Street, Orlando, FL 32806, USA; 3 Pulmonary-Critical Care, Baylor College of Medicine, 1709 Dryden Road, Suite 9.70, Houston, TX 77030, USA; 4 Pulmonary-Critical Care, Moses Cone Health System, 520 N Elam Avenue, Greensboro, NC 27403, USA; 5 Pulmonary-Critical Care, Skyline Medical Center, 3443 Dickerson Pike, Nashville, TN 37207, USA

## Abstract

**Introduction:**

Hospitalized patients are often unable to ingest or tolerate oral antipyretics and recently an aqueous formulation of intravenous (IV) ibuprofen was approved by the US-FDA for the reduction of fever in adults.

**Methods:**

We evaluated IV ibuprofen to reduce fever exceeding 101.0°F, measured as the percentage of subjects achieving a temperature <101.0°F at four hours after a single dose of IV ibuprofen vs. placebo. Secondary evaluations included the effect on temperature at 24 hours. Nine sites randomized patients to receive either a placebo or IV ibuprofen (100, 200, or 400 mg), and patients were given four hours for six doses. Subjects were excluded for platelet count <30 k and/or creatinine >3.0 mg/dL.

**Results:**

At entry, there were no significant baseline differences between the IV ibuprofen group and placebo, n = 120. At four hours, the number (percentage) with T<101.0°F was: Placebo n = 9/28 (32%); 100 mg IV ibuprofen n = 19/31 (61%), *P *= 0.0264; 200 mg IV ibuprofen n = 21/30 (70%) *P *= 0.0043; 400 mg IV ibuprofen n = 24/31 (77%) *P *= 0.0005. A total of 53/120 patients (44%) were prospectively defined as critically ill at baseline and similar temperature reductions were observed in this subgroup. There were no statistically significant differences between treatment groups or when compared to placebo in transfusion, bleeding, renal failure or mortality.

**Conclusions:**

All doses of IV ibuprofen tested reduced fever at four hours and throughout the first 24 hours of dosing. The 400 mg dose was effective in lowering temperature to normal and maintaining this over the first 24 hours of dosing. IV ibuprofen was effective in reducing fevers in critically ill and non-critically ill groups. Following 24 hours of administration of IV ibuprofen, no clinically significant differences in any safety parameter including renal function or bleeding occurred through the 28-day follow-up period.

**Trial registrations:**

Clinicaltrials.gov registration number: NCT01131000.

## Introduction

Ibuprofen is a non-steroidal anti-inflammatory drug (NSAID) that is approved as an oral treatment for mild to moderate pain and for the reduction of fever in adults and in children [1]. Ibuprofen was first developed as an antirheumatic drug in the 1960 s [2]. Ibuprofen is believed to work by inhibiting the formation of prostaglandins, thereby reducing inflammation. Studies have demonstrated the success of oral ibuprofen in the reduction of fever as well as reduction in the subjective symptoms associated with fever [3,4].

Oral ibuprofen is commonly used in hospitals to treat adult patients who develop fevers during hospitalization. However, hospitalized patients with endotracheal intubation, sedation, reduced gastric motility, nausea, recent surgery, or other factors are frequently unable to ingest, digest, absorb, or tolerate oral antipyretics. However, the US-FDA recently approved an aqueous formulation of IV ibuprofen for the reduction of fever in adults (Cumberland Pharmaceuticals, Inc., Nashville, TN, USA) to address this unmet medical need. The current study was designed to evaluate the efficacy of IV ibuprofen in patients with fever greater than or equal to 101.0°F compared to placebo.

## Materials and methods

This multicenter, randomized, double-blind, parallel, placebo-controlled clinical study was designed to assess the efficacy, safety, and pharmacokinetics of IV ibuprofen in adult subjects with fevers greater than or equal to 101.0°F. Subjects were randomized to receive a placebo or one of three doses of IV ibuprofen (100 mg, 200 mg, or 400 mg), so that each of the four treatment groups would consist of approximately 30 subjects. Randomization was performed by site and was stratified on the basis of the severity of the patient's condition. Randomization envelopes were provided to each site and were opened by the pharmacist in sequential order. At the time of randomization, at least 33% of the patients randomized were to be critically ill (in the hospital requiring mechanical ventilation for respiratory failure, pressor support for hypotension, or both), and at least 33% were to be not critically ill. Participants were assigned to treatment using a permuted block randomization scheme. A unique treatment number coupled to one of three active doses or placebo therapy was assigned to each study participant. Within any given center, treatment numbers were assigned sequentially to participants in the order they were enrolled, based upon stratification. The randomization block size was four. The randomization block size was not revealed during the course of the study to assist in maintaining the blind. The study was double-blind with respect to the treatment assignment. The patient, Investigator, and sponsor were blinded to the assigned treatment until all patients had completed the protocol and after the study database had been analyzed. Randomization codes were generated using a pseudo-random number generator and stickers for randomization envelopes were generated automatically. Each subject was assigned a unique treatment number linked to the assigned treatment number of the master randomization list. After opening the randomization envelope, provided to each site, the study site pharmacist or pharmacy technician prepared each patient's doses of IV ibuprofen or placebo and provided these to the Investigator in identical infusion bags labeled with the patient's identification number. Patients were enrolled from sites in North America, Thailand and Australia, between 2002 and 2005. Study material or placebo was administered in a 100 mL bag of normal saline. Addition of IV ibuprofen to the bag for dilution did not noticeably affect the volume of the infusion bag, since only a maximum of 4 mL was added. However, pharmacists were instructed to puncture the port of the placebo/normal saline infusion bag such that upon inspection it would appear identical to a bag prepared with IV ibuprofen.

Hospitalized patients were included if: fever developed within previous seven days, was documented by temperature >/= 101.0°F, and have IV access. Exclusions were: baseline platelet count was less than 30,000/mm^3 ^; baseline creatinine was greater than 3.0 mg/dL; <18 years old, received antipyretic drugs <4-hrs of dosing; allergy to IV ibuprofen, NSAIDs, or COX-2 inhibitors; pregnancy; history of head trauma requiring hospitalization, intracranial surgery or stroke within 30 days or history of arteriovenous malformation, cerebral aneurysm or central nervous system mass lesion; weighed <40 kg; history of bleeding diatheses, history of gastrointestinal bleeding that required medical intervention less than six weeks unless definitive surgery had been performed; required full anticoagulation or therapy with activated protein c within six hours of dosing; fever secondary to drug reaction; expected life span <14 days; required treatment with corticosteroids; neurogenic fever; required dialysis or received nephrotoxic drugs; major surgery <12 hours of dosing unless adequate hemostasis had been achieved. The preferred method of temperature measurement was core. If a non-core route was used, temperature measurement was to be verified by an additional route of measurement; the route of temperature measurement used immediately before randomization was to be the same immediately before dosing and for all temperature measurements during the treatment period. Treatment consisted of one dose of study drug every four hours, for a total of six doses. Assessments of temperature, vital signs, laboratory measurements, and safety monitoring were performed from baseline through Hour 120. Patients' hospital course was followed throughout remainder of hospitalization through Day 28 for the occurrence of serious adverse events. An equal volume of normal saline was used as placebo. The primary efficacy variable was based on the reduction in the percentage of patients with a body temperature of <101.0°F at four hours post initiation of the first dose of study drug. Safety assessments included vital signs, chemistry and hematology results, transfusion requirements and monitoring for adverse events. Blood samples were required by protocol to be collected at baseline and specific post-dosing times during the study from the first 98 subjects to measure IV ibuprofen pharmacokinetics.

The study protocol was approved by an appropriate Independent Ethics Committee (IEC) or Institutional Review Board (IRB) at each clinical study site. Consent was obtained from the patient or IRB-approved designated surrogate. This multi-center study was sponsored by Cumberland Pharmaceuticals Inc. (CPI). The authors had access to all data and prepared the manuscript. A data safety monitoring board (DSMB) reviewed serious adverse events and study results as they related to safety and quality.

### Rescue treatment for persistent fever

Rescue treatment was available to subjects who met treatment failure criteria. Treatment failure was a temperature of >/= 103.0°F during Treatment Period after a minimum of two hours post study drug or placebo. The Investigator decided whether to administer rescue treatment. Rescue treatments included acetaminophen, cold packs, cooling blankets, alcohol baths or other treatments designated by the physician, excluding aspirin or NSAID's. Delivery of study drug or placebo was discontinued for any patient receiving rescue treatment.

For randomized subjects to be given the first dose of study drug, they must have remained febrile to within 15 minutes prior to dosing of study drug.

### Statistical analysis

Data from a published study of intravenous ibuprofen in sepsis subjects were used to provide information about treatment differences in subjects treated with 800 mg intravenous ibuprofen or placebo [5]. At four hours after administration of the first dose, 78.2% of the subjects who received intravenous ibuprofen and 41.8% of the subjects who received placebo had temperatures below 101.0°F (38.3°C). On the basis of these data, a sample size of 30 subjects per treatment group in the ITT analysis would provide 80% power for a χ^2 ^test, at the significance level of ∞ = 0.05, to detect this same treatment difference approximately 37%).

The data demonstrated are for the Intention to Treat population (ITT). Continuous data were summarized by mean and standard deviation or standard error. Categorical data were summarized by frequency and the percentage of subjects in each treatment group. All statistical tests were two-sided, with *P*-values less than 0.05 for treatment differences to be considered significant. The primary endpoint was analyzed by using the Cochran-Mantel-Haenszel (*CMH*) procedure adjusted for center. An analysis of variance (ANOVA) model was used to compare differences between treatment groups in the area under the temperature versus time curve (AUC-T°) in the first 24 hours, when the AUC-T° calculated was the difference between the measured temperature at a given time point and a target (normal) temperature of 98.6°F. On the basis of plasma ibuprofen concentration-time data, pharmacokinetic parameters were assessed by using a non-compartmental model. Area under the concentration-time curve from Hour 0 to Hour 4 (AUC_0 to 4_), was calculated by using the linear-log trapezoidal rule: linear trapezoidal rule up to time to maximum concentration, and then a log trapezoidal rule for the remainder of the curve, where the last measurable time is at Hour 4.

All statistical computations were performed using SAS^®^.

## Results

Between June 2002 and August 2005, 4,465 patients were screened at 10 clinical centers, 120 of whom were enrolled in the study. Patients screened but not enrolled either met exclusion criteria or became afebrile prior to administration of the first scheduled dose of study medication. These 120 subjects were randomized into four treatment groups: 100 mg IV ibuprofen (n = 31); 200 mg IV ibuprofen (n = 30); 400 mg IV ibuprofen (n = 31) and placebo (n = 28). A total of 53 (44%) of subjects met critically ill criteria at randomization and 67 (56%) were non-critically ill. Overall, 65% of patients were enrolled from sites in the United States, 33% from Thailand and 2% from Australia, Table 1. Critically ill subjects were enrolled exclusively from US sites. Table 1 demonstrates baseline data. There were no differences among the four arms of the study in baseline demographics. Also, analyses of baseline data compared critically ill and non-critically ill populations and combined IV ibuprofen treatment groups versus placebo, and there were no statistically significant differences between the critically ill versus critically ill-placebo, non-critically ill versus non-critically ill placebo or the combined IV ibuprofen group versus the placebo group, Table 1. There were no differences in mean age, Acute Physiology and Chronic Health Evaluation II (APACHE II), or site of infection. No differences were found across critically ill groups for days of mechanical ventilation, ICU days or hospital days. Similarly, no differences were found across non-critically ill groups for hospital days, Table 2.

**Table 1:** Baseline Parameters

	100 mg IVIb (n = 31)	200 mg IVIb (n = 30)	400 mg IVIb (n = 31)	Placebo (n = 28)	Total (n = 120)
**N: Critically Ill**	14 (45%)	12 (40%)	14 (45%)	13 (46%)	53 (44%)
**N: Non-Critically Ill**	17 (55%)	18 (60%)	17 (55%)	15 (54%)	67 (56%)
**In ICU at BL**	17 (55%)	15 (50%)	17 (55%)	16 (57%)	65 (54%)
**On Mechanical Ventilation at BL**	14 (45%)	12 (40%)	14 (45%)	13 (46%)	53 (44%)
**On Presssor Support at BL**	2 (7%)	2 (7%)	0 (0%)	0 (0%)	4 (3%)
**Age (yrs)**	40.1 (± 19.0 SD)	34.5 (± 15.0 SD)	39.2 (± 17.2 SD)	37.0 (± 19.1 SD)	37.8 (± 17.5 SD)
**Gender**	23 M, 8 F	22 M, 8 F	22 M, 9 F	21 M, 7 F	88 M, 32 F
**Race**	Asian 10; Black 5; Caucasian 13; Hispanic: 2; Haitian 1.	Asian 10; Black 4; Caucasian 15; Hispanic 1; Haitian 0.	Asian 10; Black 1; Caucasian 16; Hispanic 4; Haitian 0.	Asian 10; Black 3; Caucasian 14; Hispanic: 1; Haitian 0.	Asian 40; Black 13; Caucasian 58; Hispanic 8; Haitian 1.
**Temperature (/°C)**	39.07 ± 0.61	39.07 ± 0.71	39.16 ± 0.72	38.89 ± 0.48	N/A
**Highest Temp Prior to BL (°C)**	39.4 ± 0.7 SD	39.4 ± 0.6	39.4 ± 0.7	39.2 ± 0.8	39.4 ± 0.7
**Heart Rate (bpm)**	106.0 ± 20.4	105.4 ± 18.4	102.7 ± 18.4	102.9 ± 15.4	104.3 ± 18.1
**Mean Arterial Pressure**	81.7 ± 12.9	83.3 ± 11.7	87.8 ± 12.7	88.1 ± 15.6	85.2 ± 13.4
**Height** **(cm)**	170.3 ± 10.3	170.1 ± 10.5	168.2 ± 10.1	168.8 ± 11.8	169.4 (±10.6 SD)
**Weight** **(kg)**	77.9 ± 32.6	80.3 ± 29.2	72.5 ± 23.4	78.4 ± 26.6	77.2 (± 28.0)
**Positive Blood Culture**	13 (42%) (8 ± Malaria)	13 (43%) (10 ± Malaria)	12 (39%) 9 ± Malaria	13 (46%) 10 ± Malaria	51 (43%) 37 ± Malaria
**Modified APACHE II**	11.4 (± 7.6 SD)	12.0 (± 7.7 SD)	13.4 (± 7.8 SD)	12.5 (± 9.7 SD)	12.3 (± 8.1 SD)
**Glasgow Coma Score**	3 to 8: 4 (13%) 9 to 12: 3 (10%) >13:24 (77%)	3 to 8: 6 (20%) 9 to 12: 3 (10%) >13:21 (70%)	3 to 8: 7 (23%) 9 to 12: 4 (13%) >13:20 (65%)	3 to 8: 5 (18%) 9 to 12: 3 (11%) >13:20 (71%)	3 to 8: 22 (18%) 9 to 12: 13 (11%) >13:85 (71%)
**Emerg. PostOp** **Elect Post Op** **Non Operative**	2 (6%) 0 (0%) 29 (94%)	4 (13%) 0 (%) 26 (87%)	2 (6%) 3 (10%) 26 (84%)	1 (4%) 0 (0%) 27 (96%)	9 (%) 3 (%) 108 (%)
**Chronic Conditions** **(from Modified APACHE II)**	Liver 0(0%) CV 0 (0%) Resp 1(3%) Renal 0(0%) Immun 2(6%) None 28(90%)	Liver 0(0%) CV 0 (0%) Resp 1(3%) Renal 0 (0%) Immun 2(7%) None 27(90%)	Liver 0 (0%) CV 0 (0%) Resp 1(3%) Renal 0 (0%) Immun1(3%) None 29 (94%)	Liver 0 (0%) CV 0 (0%) Resp 1 (4%) Renal 0 (0%) Immun (7%) None 25 (9%)	Liver 0 0%) CV 0 (0%) Resp 4(3%) Renal 0 (0%) Immun 7(6%) None109 (91%)
**WBC**	11.6 ± 7.2	10.9 ± 6.6	9.6 ± 5.0	11.6 ± 6.4	10.9 ± 6.3
**Creatinine (mg/dL)**	0.95 ± 0.26	.97 ± 0.25	0.97 ± 0.35	1.0 ± 0.44	0.97 (±0.33)
**Hematocrit** **(%)**	36.8 ± 7.7	34.1 ± 6.5	33.7 ± 6.0	34.5 ± 6.9	34.8 ± 6.8
**Hemoglobin (g/dL)**	12.3 ± 2.5	11.4 ± 2.2	11.4 ± 2.1	11.6 ± 2.3	11.7 ± 2.3
**Platelet Count**	221.6 (± 158.7)	189.7 (± 111.3)	173.6 (±116.3)	220.0 (±123.61)	200.9 (± 129.1)
**Probable Site Infection**	None 1(3%); Blood 9 (29%); Lung 9 (29%); Abdom 2 (6%) Other 3 (10%) CNS 2 (6%) Urine Tr 0 (0%) Skin/Tis 2 (6%) Up Resp 2(6%) GYN 0 (0%) IV 1(3%) Unk 2(6%)	None (3%) Blood 10 (33%) Lung 7 (23%) Abdom 4 (13%) Other 3 (10%) CNS 3 (10%) Urine Tr 0(0%) Skin/Tis1 (3%) Up Resp 0(0%) GYN 0 (0%) IV 0 (0%) Unk 1 (3%)	None 2 (6%) Blood 10 (32%) Lung 9 (29%) Abdom 1 (3%) Other 4 (13%) CNS 1 (3%) Urine Tr 3 (10%) Skin/Tis 0 (0%) Up Resp1 (3%) GYN 0(0%) IV 0(0%) Unk 0(0%)	None 2 (7%) Blood 11 (39%) Lung 5 (18%) Abdom 4 (14%) Other 1 (4%) CNS 0 (0%) Urine Tr 2 (7%) Skin/Tis1(4%) Up Resp 0 (0%) GYN 1 (4%) IV 0(0%) Unk 1(4%)	None 6(5%) Blood 40 (33%) Lung 30 (25%) Abdom 11 (9%) Other 11 (9%) CNS 6(5%) Urine Tr 5 (4%) Skin/Tis 4 (3%) Up Resp 3 (3%) GYN (1%) IV 1(1%) Unk 2(2%)

BL, Baseline.

**Table 2:** Ventilation and length of hospital stay

	Placebo	100 mg	200 mg	400 mg
Critically ill patients				
# patients	13	14	12	14
				
Mechanical ventilation				
median days	14	14	11	11
				
ICU length of stay				
median days	18	14	15	12
				
Hospital length of stay				
median days	18	35	25	20
				
Non-critically ill patients				
# patients	15	17	18	17
				
Hospital length of stay				
median days	29	14	23	29

**Figure 1:**
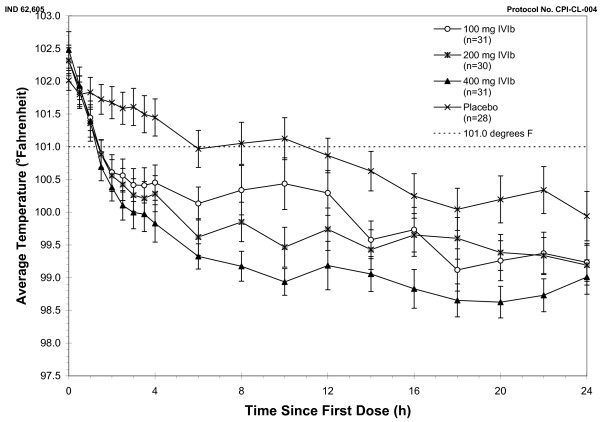
**Temperature (°F) by treatment, ITT population**. ITT, intention to treat; IVIb, intravenous ibuprofen.

**Figure 2:**
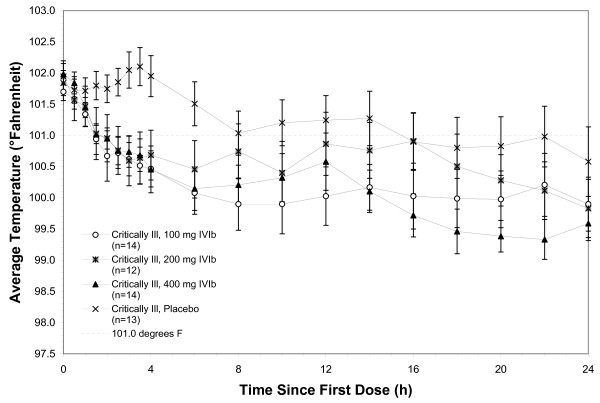
**Temperature over time, critically ill**.

**Figure 3:**
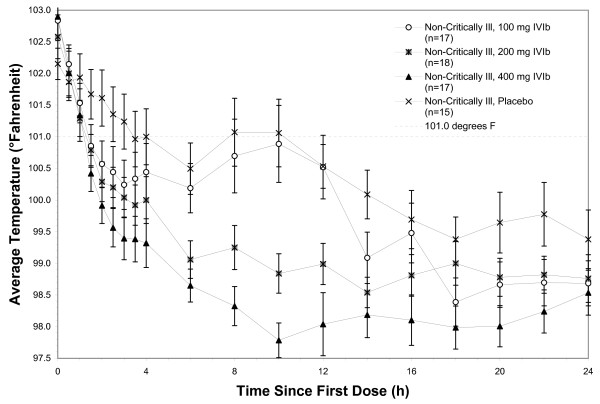
**Temperature over time, non-critically ill**.

In the Intention to Treat (ITT) population, fever at four hours in the 400 mg IV ibuprofen group (primary endpoint) showed 24 of 31 (77%) of subjects, compared to 9 of 28 (32%) of the placebo group with a temperature <101.0°F, *P *= 0.0005. For secondary endpoints, 21 of 30 (70%) of subjects in the 200 mg IV ibuprofen group, and 19 of 31 (61%) of subjects in the 100 mg IV ibuprofen group, compared to 9 of 28 (32%) of the placebo group had a temperature <101.0°F, *P *= 0.0264. Figure 1 demonstrates the mean temperatures of study subjects by protocol arm in the first 24 hours. Decrements in temperature were observed in both the critically ill subjects receiving IV ibuprofen vs. critically ill subjects receiving placebo and in the non-critically ill subjects receiving IV ibuprofen vs. non-critically ill subjects receiving placebo, Figures 2 and 3, respectively.

In a secondary analysis of efficacy, the efficacy of IV ibuprofen was analyzed by assessing the percentage of treatment failures compared to placebo. In the ITT population at 24 hours, the mean time to treatment failure in hours was: Placebo 5.70 +/- 1.97; 100 mg IV ibuprofen, 7.39 +/- 1.39, *P *= 0.75 compared to placebo; 200 mg IV ibuprofen, 10.29 +/- 3.25, *P *= 0.22 compared to placebo; 400 mg IV ibuprofen, 10.75 +/- 2.75, *P *= 0.39 compared to placebo.

In the ITT by 24 hours, the mean temperature decrease from baseline was: Placebo 2.07°F +/- 2.37; 100 mg IV ibuprofen, 3.07°F +/- 1.95°F with a mean difference of 1.0°F (-4.77 to 6.75, 95% CI); 200 mg IV ibuprofen, 3.12°F +/- 1.88°F with a mean difference of 4.40°F (-1.40 to 10.21, 95% CI); 400 mg IV ibuprofen, 3.45°F +/- 2.02 with a mean difference of 1.37°F (-4.39 to 7.13, 95% CI). The area under the temperature versus time curve (AUC-T) was calculated for 0 to 24 hours, for temperature above 98.4°F. For the ITT, comparison of treatment group against placebo showed that AUC-T over 24 hours was significantly less for all active treatment groups compared to the placebo group, with average reductions of 15.30°F deg*h (100 mg IV ibuprofen), 21.46°F deg*h (200 mg IV ibuprofen) and 28.97°F deg*h (400 mg IV ibuprofen).

The pharmacokinetics of IV ibuprofen were determined. The AUC_0-4 _was approximately dose proportional for the 200 mg and 400 mg dose levels of IV Ibuprofen, see Table 3.

**Table 3:** Area under the curve (AUC 0-4) for plasma ibuprofen concentrations

Average of treatment Group	100 mg IV Ibuprofen	200 mg IV Ibuprofen	400 mg IV Ibuprofen
AUC0-4 (μg.h/mL)	22.33 +/- 12.75 (SD)	32.62 +/- 17.39 (SD)	70.64 +/- 31.93 (SD)

**Figure 4:**
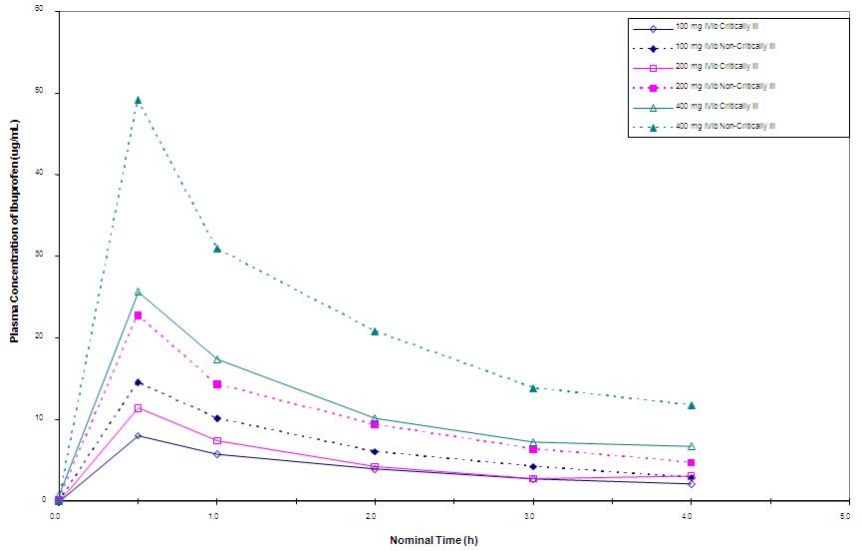
**Mean ibuprofen concentrations (hour 0 to 4), by treatment group and randomization stratum**.

Figure 4 demonstrates the mean plasma concentrations of ibuprofen for each of the three drug dosage arms in both the critically ill and non-critically ill populations. There is a numerically lower concentration within the critically ill population at each dose compared to the same dose delivered to the non-critically ill population. Interestingly, decrements in temperature within the critically ill were less for each dose as compared to the decrements in fever for the non-critically ill population (Figures 2 and 3, respectively).

### Safety

Exposure to the study drug among the 120 subjects included: 27 of the 31 subjects (87%) in the 100 mg IV ibuprofen treatment group received all six doses of the study drug, all 30 subjects (100%) in the 200 mg IV ibuprofen treatment group received all six doses of the study drug, 28 of the 31 subjects (90%) in the 400 mg IV ibuprofen treatment group received all six doses of the study drug, and 23 of the 28 (82%) in the placebo group received all six doses of the placebo.

There were no differences in serious adverse events (SAEs) or adverse events (AEs) between subjects treated with IV ibuprofen and placebo, particularly in regards to bleeding or renal failure, except for bacteremia. There were four patients post-enrollment in the 100 mg IV ibuprofen and one patient in the 200 mg IV ibuprofen groups with bacteremia; no bacteremia was reported in the 400 mg IV or placebo groups (*P *= 0.045). Site investigators reported these bacteremias as consistent with underlying disease and not related to study drug. There were 17 subjects for whom 29 SAEs were reported: 100 mg IV ibuprofen six subjects (five critically ill subjects, one non-critically ill); 200 mg IV ibuprofen five subjects (four critically ill, one non-critically ill); 400 mg IV ibuprofen three subjects (two critically ill, one non-critically ill) and placebo three (two critically ill, one non-critically ill), Table 4. Of the 29 events, none were felt by the site investigator to be related to the study drug. Choices of relationship to study material could have been coded as related, not related and unknown/uncertain. Of the 29 events, none were coded as related; 24 were coded as not related; and 5 as unknown/uncertain (serious AE's 100 mg IVIb three; 400 mg IVIb one and placebo one). Those five events were: dilated and fixed right pupil, punctuate intraparenchymal hemorrhage, left humerus fracture, multi-system organ failure secondary to sepsis, bilateral tension pneumothorax. There were no statistically significant differences between treatment groups with regards to the requirements for transfusion of red cell units (100 mg IV ibuprofen five; 200 mg IV ibuprofen three; 400 mg IV ibuprofen five and placebo four). There were no patient deaths during the 24-hour study treatment period. Six subjects participating in this study (5%) died during the 28-day study observation period from conditions related to their critical medical condition at the time of enrollment in the study.

**Table 4:** Adverse events and serious adverse events

Number of subjects (% of treatment group)	100 mg IV Ibuprofen		200 mg IV Ibuprofen		400 mg IV Ibuprofen		Placebo	
Subjects experiencing no AEs	4	13%	5	17%	8	26%	3	11%
Subjects experiencing any AEs (including SAEs)	27	87%	25	83%	23	74%	25	89%
Subjects experiencing SAEs (including death)	6	19%	5	17%	4	13%	4	14%
Patient deaths	1	3%	2	7%	2	6%	1	4%
Total subjects	31		30		31		28	

The laboratory parameters with the most subjects experiencing changes from not clinically significant (NCS) at baseline to clinically significant (CS) at 24 hours post-dose were: potassium (8 subjects), albumin (8 subjects), protein (9 subjects), LDH (6 subjects); hemoglobin (10 subjects), eosinophils (12 subjects), WBC (6 subjects), HCT (6 subjects), platelets (5 subjects), monocytes (4 subjects), lymphocytes (6 subjects) and PMN's + bands (5 subjects). There was no effect related to treatment. There were no subjects who experienced renal complications or a rise in creatinine, a change in hemoglobin related to IV ibuprofen, and no subjects experienced a change in platelet count that was thought to be related to IV ibuprofen, Tables 5, 6 and 7.

**Table 5:** Creatinine values over time with actual and percent change from baseline by treatment

**Creatinine **(mg/dL)		Lab values over time				Change from baseline (actual and percent)					
			
		Baseline	Hour 24	Hour 72	Hour 120	to Hour 24		to Hour 72		to Hour 120	
**100 mg IV Ibuprofen**	Average	0.95	0.85	0.85	0.87	-.009	-6.4%	-0.09	-4.1%	-0.08	-1.9%
	SD	0.26	0.21	0.21	0.30	0.23	25.6	0.33	37.0	0.40	48.7
	N	31	30	29	28	30	30	29	29	28	28
	Min	0.57	0.50	0.50	0.50	-0.60	-44.4%	-0.80	-50.0%	-0.90	-56.3%
	Max	1.60	1.40	1.70	2.20	0.50	71.4%	1.00	142.9%	1.50	214.3%

**200 mg IV Ibuprofen**	Average	0.97	0.85	0.90	0.83	-0.12	-11.4%	-0.07	-6.0%	-0.08	-7.5%
	SD	0.25	0.28	0.29	0.19	0.20	18.3	0.23	19.5	0.17	18.7
	N	30	30	27	24	30	30	27	27	24	24
	Min	0.60	0.11	0.50	0.50	-0.97	-89.8%	-0.60	-37.5%	-0.40	-28.6%
	Max	1.60	1.50	1.70	1.30	0.20	15.4%	0.50	41.7%	0.30	50.0%

**400 mg IV Ibuprofen**	Average	0.97	0.97	1.07	1.05	0.00	-2.8%	0.11	6.7%	0.06	5.8%
	SD	0.35	0.59	1.05	0.93	0.37	31.7	0.93	88.7	0.86	84.5
	N	31	30	31	27	30	30	31	31	27	27
	Min	0.40	0.30	0.30	0.30	-0.31	-34.1%	-0.30	-30.0%	-0.40	-33.3%
	Max	2.10	3.20	5.81	5.28	1.39	136.3%	4.79	469.6%	4.26	417.6%

**Placebo**	Average	1.00	0.94	0.86	0.87	-0.06	-5.4%	-0.14	-10.3%	-0.16	-9.8%
	SD	0.44	0.40	0.25	0.23	0.13	12.4	0.26	15.5	0.34	20.4
	N	28	28	28	24	28	28	28	28	24	24
	Min	0.60	0.42	0.50	0.50	-0.30	-33.3%	-1.10	-46.7%	-1.30	-53.3%
	Max	2.80	2.50	1.70	1.50	0.20	18.2%	0.20	25.0%	0.30	37.5%

**Table 6:** Hemoglobin values over time with actual and percent change from baseline by treatment

**Hgb **(g/dL)		Lab values over time				Change from baseline (actual and percent)					
			
		Baseline	Hour 24	Hour 72	Hour 120	to Hour 24		to Hour 72		to Hour 120	
**100 mg IV Ibuprofen**	Average	12.3	11.2	11.2	11.0	-1.1	-8.8%	-1.3	-8.2%	-1.3	-8.8%
	SD	2.5	2.1	1.6	1.8	1.0	7.3	2.0	15.3	2.0	15.9
	N	31	30	28	27	30	30	28	28	27	27
	Min	8.2	6.4	8.2	7.2	-4.0	-23.1%	-6.4	-37.0%	-7.1	-47.0%
	Max	17.3	15.1	15.3	14.9	0.6	4.6%	3.4	35.1%	3.1	37.8%

**200 mg IV Ibuprofen**	Average	11.4	10.4	10.4	10.5	-1.0	-8.5%	-1.0	-7.9%	-0.8	-5.5%
	SD	2.2	2.0	1.8	2.0	1.0	8.1	1.3	10.4	1.4	13.2
	N	30	30	26	24	30	30	26	26	24	24
	Min	7.2	6.0	6.5	6.3	-3.3	-22.7%	-4.3	-28.9%	-3.4	-24.1%
	Max	15.2	14.0	14.6	14.4	0.6	6.2%	1.1	12.7%	2.3	25.3%

**400 mg IV Ibuprofen**	Average	11.4	10.3	10.2	10.4	-1.1	-8.9%	-1.2	-9.6%	-0.8	-5.3%
	SD	2.1	1.7	1.8	1.2	1.0	8.5	1.5	12.9	1.7	14.4
	N	31	30	31	27	30	30	31	31	27	27
	Min	7.9	7.2	5.6	8.2	-2.8	-22.6%	-4.1	-39.8%	-4.2	-28.0%
	Max	16.2	14.7	13.2	13.0	1.3	15.3%	2.0	25.0%	2.5	31.3%

**Placebo**	Average	11.6	10.7	10.4	10.4	-0.9	-6.9%	-1.3	-9.6%	-1.3	-9.1%
	SD	2.3	1.9	1.7	1.7	1.2	12.4	1.5	13.8	1.7	17.8
	N	28	28	27	24	28	28	27	27	24	24
	Min	6.0	7.6	7.6	8.1	-3.0	-26.9%	-3.7	-27.8%	-4.4	-33.1%
	Max	16.2	14.6	13.3	13.8	2.5	41.7%	1.7	28.3%	3.1	51.7%

**Table 7:** Platelet values over time with actual and percent change from baseline by treatment

**Platelets **(×10^9^/L)		Lab values over time				Change from baseline (actual and percent)					
			
		Baseline	Hour 24	Hour 72	Hour 120	to Hour 24		to Hour 72		to Hour 120	
**100 mg IV Ibuprofen**	Average	222	210	270	325	-14	-7%	40	41%	112	109%
	SD	159	164	157	191	43	17	92	69	145	140
	N	31	30	28	27	30	30	28	28	27	27
	Min	41	36	73	39	-99	-41%	-191	-38%	-169	-77%
	Max	659	756	690	933	103	20%	230	269%	473	513%

**200 mg IV Ibuprofen**	Average	190	201	293	416	11	7%	101	82%	228	188%
	SD	111	127	190	239	63	27	148	99	194	180
	N	30	30	26	24	30	30	26	26	24	24
	Min	35	29	102	148	-197	-56%	-200	-57%	3	2%
	Max	477	492	965	1258	205	71%	678	412%	971	686%

**400 mg IV Ibuprofen**	Average	174	162	229	308	-9	-6%	55	54%	136	156%
	SD	116	110	126	149	36	23	103	69	168	161
	N	31	30	31	27	30	30	31	31	27	27
	Min	34	20	39	117	-87	-49%	-209	-41%	-301	-58%
	Max	516	464	562	866	115	64%	367	243%	685	533%

**Placebo**	Average	220	213	268	339	-7	-4%	43	32%	127	98%
	SD	124	136	137	137	61	28	84	56	114	122
	N	28	28	27	24	28	28	27	27	24	24
	Min	48	5	22	61	-163	-97%	-192	-87%	-162	-64%
	Max	541	448	502	594	130	44%	178	213%	301	521%

## Discussion

In this population of hospitalized patients a significantly greater proportion of subjects experienced a lowering of their temperature to less than 101°F within four hours after one dose of 400 mg of IV ibuprofen as compared to those receiving the placebo, demonstrating the efficacy of IV ibuprofen to control fever in both acutely and critically ill hospitalized participants. Safety evaluations indicate that for this duration of exposure to IV ibuprofen, there were no clinically significant nephrotoxic effects of IV ibuprofen in the 168 hours after dosing or through study Day 28 in either the critically ill or in the non-critically ill cohorts.

Ibuprofen concentrations in the critically ill group were on average lower at the same time points and doses as compared to the non-critically ill group. The magnitude of temperature reduction and the percent of patients achieving temperature less than 101°F was less in the critically ill group compared to the non-critically ill group.

Controversy exists as to the clinical value of fever reduction. Patient comfort and avoidance of the adverse effects of fever, particularly when very high, are typically the goals of fever reduction. Adverse effects of fever can include discomfort, dehydration, febrile seizures in children, and increased oxygen demand [6,7]. In hospital practice, it is common to allow as needed orders for temperature reduction therapies when the patient's body temperature rises above 101°F. When patients may receive an oral agent, an antipyretic therapy is usually considered such as oral or rectal acetaminophen or use of a non-steroidal agent. In patients unable to tolerate oral therapy, the principle options are cooling blankets or other physical methods [6,7].

Fever reduction was demonstrated in each of the dose groups of IV ibuprofen with statistically significant different percentages of subjects achieving a temperature below 101°F as compared to the placebo group. Additionally, for the end points of fever reduction at 4 and 24 hours, both the critically ill and non-critically ill groups achieved a statistically significant percentage of patients achieving a temperature less than 101°F as compared to the percent of critically ill and non-critically ill subjects, respectively, who received the placebo.

This study demonstrated that IV Ibuprofen did not alter renal function. There were no significant differences in serial measures of serum creatinine or in the need for hemodialysis in either the critically ill subjects or the non-critically ill subjects. As assessed by serial measures of hemoglobin, need for transfusions of platelets and need for red cells, there was no indication that IV ibuprofen was associated with bleeding in either the critically ill or non-critically ill groups as compared to the placebo group.

This safety pattern has been described for IV ibuprofen in other hospital settings. Bernard *et al. *performed a randomized, double-blind, placebo-controlled trial of IV ibuprofen (10 mg per kilogram of body weight (maximal dose, 800 mg), given every six hours for eight doses) in 455 patients who had sepsis, defined as fever, tachycardia, tachypnea, and acute failure of at least one organ system [5]. Serum creatinine and urinary output were measured serially over a five-day period to evaluate the effects of the study treatment on renal function, and no significant differences were detected between the groups. Hemodialysis was required for the first time in 6 of 224 ibuprofen-treated patients (3 percent) and 13 of 231 placebo-treated patients (6%, *P *= 0.11). Gastrointestinal bleeding was reported in nine patients in the ibuprofen group and 16 patients in the placebo group.

## Limitations

One limitation of this trial is the relatively small numbers of critically ill and non-critically ill patients studied. In addition, the duration of exposure was relatively short but none-the-less consistent with the notion that the IV form of ibuprofen would be used only for brief periods in hospitalized patients temporarily unable to tolerate oral medications. In practice, however, patients with prolonged febrile illness, particularly those in the ICU setting, may demonstrate a need for fever reduction strategies for several days.

## Conclusions

All doses of IV ibuprofen tested reduced fever at four hours and throughout a 24-hour dosing period. Only the 400 mg dose was effective in lowering and maintaining temperature to the normothermic range over the 24-hour period of observation. IV ibuprofen was effective in reducing fevers both in critically ill and non-critically ill groups but was slightly more effective in the latter. We found no clinically significant differences in renal function, bleeding or need for renal replacement therapy or transfusions through study Day 28 in those receiving IV ibuprofen vs. those receiving the placebo. Thus, IV ibuprofen appears to be safe and effective for the short term treatment of fever in hospitalized patients unable to tolerate oral medications.

## Key messages

• An aqueous formulation of IV ibuprofen has been approved by the US-FDA for the reduction of fever in patients that cannot take oral medications; an unmet medical need.

• This study compared three different doses of IV ibuprofen to compare their effectiveness against placebo to reduce fever in hospitalized ICU and non-ICU patients with fever.

• This study demonstrated that IV ibuprofen given every four hours for six doses performed better than a placebo in reducing fever without increase in creatinine or reduction in haemoglobin.

• Many practitioners choose to relieve fever as a comfort. For those patients who have fever but may not be able to take medication by mouth, the IV preparation of ibuprofen may be appropriate for short-term use.

## Abbreviations

AEs: adverse events; AUC-T°: area under the temperature versus time curve; CMH: Cochran-Mantel-Haenszel; CPI: Cumberland Pharmaceuticals Inc; DSMB: data safety monitoring board; IEC: Independent Ethics Committee; IRB: Institutional Review Board, ITT: Intention to Treat; IV: intravenous; NSAID: non-steroidal anti-inflammatory drug; SAEs: serious adverse events.

## Competing interests

In the past five years, Dr. Morris has served on a DSMB for other studies sponsored by Cumberland Pharmaceuticals, Inc. and received $1500.00 US per year for two years of work on the DSMB.

## Authors' contributions

PM, JP, KG, PW and MA made substantial contributions to the conception and design of the study, acquisition of data and the interpretation of data. PM and PW drafted the manuscript and PM, JP, KG, PW and MA revised it critically for important intellectual content. PM, JP, KG, PW and MA have given final approval of the version to be published.
